# Gene expression changes induced by the tumorigenic pyrrolizidine alkaloid riddelliine in liver of Big Blue rats

**DOI:** 10.1186/1471-2105-8-S7-S4

**Published:** 2007-11-01

**Authors:** Nan Mei, Lei Guo, Ruqing Liu, James C Fuscoe, Tao Chen

**Affiliations:** 1 Division of Genetic and Reproductive Toxicology, National Center for Toxicological Research, FDA, Jefferson, AR 72079, USA; 2 Division of Systems Toxicology, National Center for Toxicological Research, FDA, Jefferson, AR 72079, USA; 3 Division of Personalized Nutrition and Medicine, National Center for Toxicological Research, FDA, Jefferson, AR 72079, USA; 4 School of Public Health, Sun Yat-sen University, Guangzhou 510089, P. R. China

## Abstract

**Background:**

Pyrrolizidine alkaloids (PAs) are probably the most common plant constituents that poison livestock, wildlife, and humans worldwide. Riddelliine is isolated from plants grown in the western United States and is a prototype of genotoxic PAs. Riddelliine was used to investigate the genotoxic effects of PAs via analysis of gene expression in the target tissue of rats in this study. Previously we observed that the mutant frequency in the liver of rats gavaged with riddelliine was 3-fold higher than that in the control group. Molecular analysis of the mutants indicated that there was a statistically significant difference between the mutational spectra from riddelliine-treated and control rats.

**Results:**

Riddelliine-induced gene expression profiles in livers of Big Blue transgenic rats were determined. The female rats were gavaged with riddelliine at a dose of 1 mg/kg body weight 5 days a week for 12 weeks. Rat whole genome microarray was used to perform genome-wide gene expression studies. When a cutoff value of a two-fold change and a *P*-value less than 0.01 were used as gene selection criteria, 919 genes were identified as differentially expressed in riddelliine-treated rats compared to the control animals. By analysis with the Ingenuity Pathway Analysis Network, we found that these significantly changed genes were mainly involved in cancer, cell death, tissue development, cellular movement, tissue morphology, cell-to-cell signaling and interaction, and cellular growth and proliferation. We further analyzed the genes involved in metabolism, injury of endothelial cells, liver abnormalities, and cancer development in detail.

**Conclusion:**

The alterations in gene expression were directly related to the pathological outcomes reported previously. These results provided further insight into the mechanisms involved in toxicity and carcinogenesis after exposure to riddelliine, and permitted us to investigate the interaction of gene products inside the signaling networks.

## Background

Pyrrolizidine alkaloids (PAs) are common constituents of thousands of plant species around the world and PA-containing plants are probably the most common poisonous plants affecting livestock, wildlife, and humans. Thus, the human health risk posed by exposure to PAs has been a concern [1,2]. Out of more than 6000 plants, about 660 PAs and their *N*-oxide derivatives have been identified, and at least half of them are genotoxic and many are tumorigenic [1-4]. Riddelliine is a representative genotoxic PA, and is present in plants growing in the rangelands of the western United States [5-7]. These plants containing riddelliine appear to enter the human food chain since riddelliine residues have been detected in meat, milk, and honey [7]. Riddelliine was nominated by the U.S. Food and Drug Administration to the National Toxicology Program (NTP) for genotoxicity and carcinogenicity testing due to the potential for human exposure [5].

Riddelliine is a 12-membered macrocyclic diester PA with an α, β-unsaturated double bond linked to the ester group at the C-7 position of the necine base. Riddelliine is completely absorbed within 30 minutes after gavage dosing to rodents and metabolized to the major metabolites, 6,7-dihydro-1-hydroxymethyl-5*H*-pyrrolizine (DHP) and riddelliine *N*-oxide, by mammalian microsomes [8-10]. ^32^P-Postlabeling-HPLC analysis has identified a set of DHP-derived DNA adducts from rat and human liver microsomal metabolism of riddelliine in vitro [11] and in the livers of rats treated in vivo [12]. A linear dose-dependent formation of DHP-derived DNA adducts was observed in riddelliine-treated rats [12,13]. Riddelliine is genotoxic both in vitro and in vivo, inducing increases in sister chromatid exchange, chromosomal aberrations, unscheduled DNA synthesis, and micronucleated erythrocyte frequencies [6]. In the NTP carcinogenicity studies, riddelliine was tumorigenic, causing liver tumors in male mice and both sexes of rats, mononuclear cell leukemia in rats, and lung neoplasms in female mice. Riddelliine induced a high incidence of liver hemangiosarcomas (derived from endothelial cells) and lower incidences of hepatocellular carcinoma (HCC; derived from parenchymal cells) in rat liver [5,6].

In our previous studies, we observed that riddelliine is mutagenic in the liver of riddelliine-treated rats and that the mutant frequencies (MFs) increased in a linear dose-dependent manner. Riddelliine also produced a unique mutational spectrum in the liver *cII *gene of Big Blue rats with G:C → T:A transversions being the major type of mutation [14]. Moreover, we found that the *cII *MF in liver endothelial cells from riddelliine-treated rats was significantly greater than the *cII *MF in endothelial cells from control rats, suggesting that the relatively high mutagenicity of riddelliine in rat liver endothelial cells may be partially responsible for the tumorigenic specificity of this agent [15]. It has been reported that riddelliine-treated mice and rats have higher and more persistent DNA adduct levels in liver endothelial cells than in parenchymal cells [9]. The sensitivity of tissues and cell types to the mutagenicity of carcinogens may be an important factor in the tissue- and cell-specificity of tumorigenesis.

Microarray technology has a profound impact on gene expression research because of its ability to examine the expression of thousands of genes at a time. The differentially expressed genes that are identified may be used to develop potential biomarkers, elucidate molecular mechanisms, and create gene signatures that identify classes of samples [16]. This technology, as one of the core technologies for pharmacogenomics and toxicogenomics, provides new insights into the effects of botanical chemicals on biological systems and allow the macrodissection of molecular events in botanical carcinogenesis [17,18]. Identification of unique gene expression patterns produced by botanical carcinogens may allow us to elucidate the mechanisms of action. In this study, we treated rats with a tumorigenic dose of riddelliine and conducted microarray analysis of gene expression in the target tissue liver. We found that the gene expression profiles were significantly altered by riddelliine treatment, and many of the differentially expressed genes were involved in metabolism, injury of liver endothelial cells, liver abnormalities, and cancer development.

## Results and discussion

### Samples and DNA microarray data analysis

Liver samples used in this study were from a previous report [14] in which female Big Blue rats were treated with a carcinogenic dose (1 mg/kg, 5 days per week) of riddelliine for 12 weeks. The MF in the liver *cII *gene was about 3-fold higher than those in the untreated group and the mutation spectra in the riddelliine-treated rats was significantly different from those in the control rats. In the present study, gene expression profiles were determined for the livers of control and riddelliine-treated rats using the Applied Biosystems' Rat Genome Survey Microarray which contains 26,857 verified rat genes. Because liver tumors had not developed at the 12 week sacrifice time, the gene expression changes reflected early events in the carcinogenesis process.

**Figure 1:**
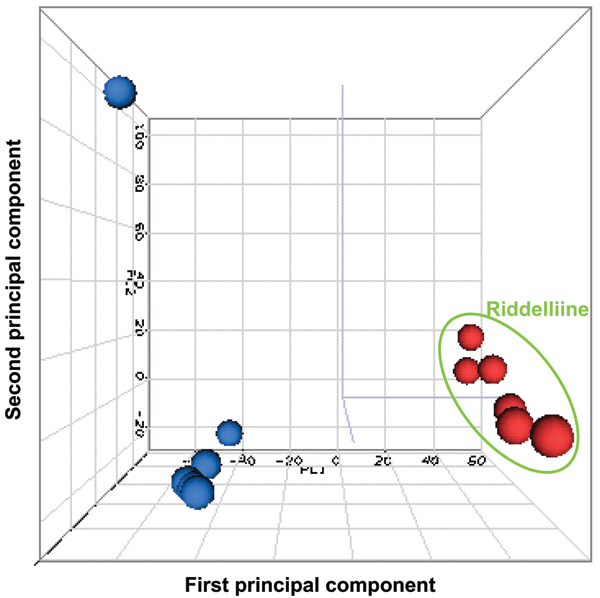
**Principal component analysis of gene expression profiles from livers of control and riddelliine-treated rats**. No specific cut off was applied and the intensity of whole rat genome data was used. The blue and red dots indicate control and riddelliine-treated samples, respectively.

**Figure 2:**
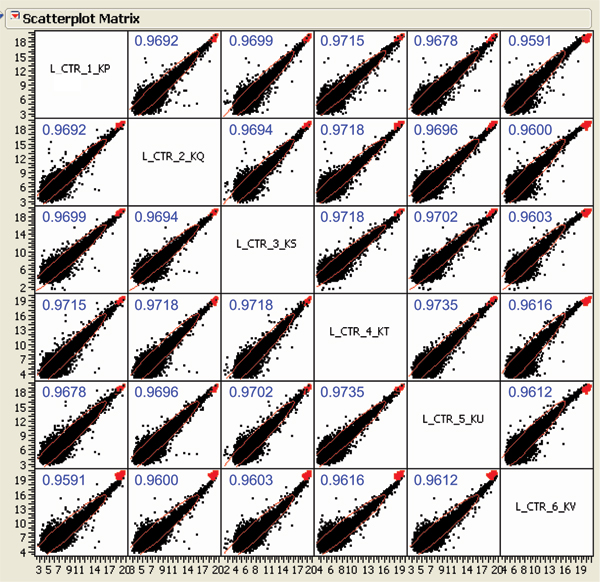
**The pair-wise log2 intensity correlations within six samples of control group**. No specific cut off was applied and the intensity of whole rat genome data was used. "L_CTR" means liver control, and R values are shown.

**Figure 3:**
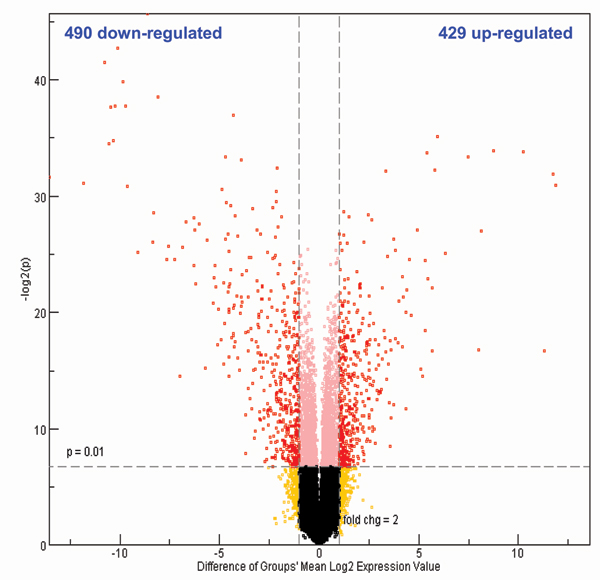
**Volcano plots (log2 fold change vs. -log2 *P*-value)**. A gene was identified as significantly changed if the fold change was greater than 2 (up or down) and the *P*-value was less than 0.01 in comparison to the control group (red symbols). Each group consisted of 5 or 6 replicates.

**Figure 4:**
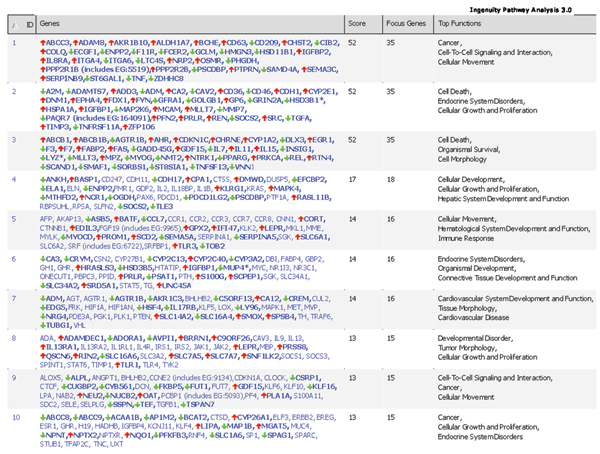
**The pathway analysis of gene expressions for the liver of rats treated with riddelliine**. The top 10 networks were selected by Ingenuity pathway analysis database.

After data normalized by Quantile normalization which is recommended by the manufacturer, the intensities of the whole rat gene data were analyzed by Principal Components Analysis (PCA, Figure 1). A separation between control and riddelliine-treated groups was observed, indicating that there was a riddelliine-treatment effect on liver gene expression. PCA analysis also demonstrates that sample #6 from the control group appears to be different from the rest of the five sample arrays. Figure 2 shows the pair-wise log2 intensity Pearson's correlation coefficients for the 6 controls samples. The correlation coefficient numbers containing samples #6 were lower than the others. Therefore, this array was excluded from further data analysis. Differentially expressed genes were identified based on the criteria of fold-change greater than 2 (up and down) and *P*-value less than 0.01 in comparison to the control group (Figure 3). A total of 919 genes satisfied the requirements, of which 429 genes were up-regulated and 490 genes were down-regulated in response to riddelliine treatment. Among the differentially expressed genes, 781 were in the Ingenuity Pathway Analysis (version 3.0) database, and 238 genes were mapped to the networks. Figure 4 shows the top 10 networks; each network was associated with specific genes and involved in different functions. The major relevant functions altered by riddelliine treatment in rat liver are listed in Table 1. These functions include cancer, cell death, tissue development, cell morphology, cell-to-cell signaling and interaction, and cellular development. Because riddelliine treatments induce relatively high DNA adduct formation, mutation induction, and tumor incidence in the liver endothelial cells [6,9,14,15], we focused our analysis on genes involved in carcinogenesis, mainly metabolism, injury of endothelial cells, liver abnormalities, and cancer development using Ingenuity Pathway Analysis.

**Table 1:** The major relevant functions altered by riddelliine treatment in liver

**Function Category**	**Significance**	**Associated Genes**
Tissue Development	2.59E-5 – 4.05E-2	64
Cell Morphology	3.35E-5 – 3.99E-2	64
Cancer	8.84E-5 – 3.99E-2	70
Cell Death	8.84E-5 – 3.99E-2	66
Cell-To-Cell Signaling and Interaction	5.51E-4 – 4.05E-2	58
Lipid Metabolism	7.31E-4 – 3.99E-2	44
Cellular Development	2.12E-3 – 3.56E-2	58
Cellular Growth and Proliferation	2.12E-3 – 3.99E-2	37
Cell Cycle	3.96E-3 – 4.04E-2	31
Cellular Movement	4.00E-3 – 3.89E-2	39

### Alteration of metabolizing genes

Since metabolic activation of riddelliine is required for liver tumor induction [12,19], we investigated the gene expression changes of drug metabolizing genes. Table 2 shows phases I, II, and III drug metabolizing genes whose expression was significantly changed by riddelliine treatment. Four phase 1 cytochrome P450 genes (Cyp2c12, Cyp2e1, Cyp3a9, and Cyp26) were up-regulated. Phase 2 glutathione *S*-transferase (Gsta3) and phase 3 ATP-binding cassette transporters (Abcb1a and Abcc3) were also up-regulated. In addition, there were many down-regulated genes involved in these 3 subgroups (Table 2).

**Table 2:** Genes involved in drug metabolisms altered by riddelliine treatment in liver

**Gene symbol**	**Gene description**	**Locus link ID**	**Fold change**	***P*-value**
*Phase I metabolism*				
CYP2C	cytochrome P450, family 2, subfamily c	29277	0.003	0.00000
CYP2C12	cytochrome P450, family 2, subfamily c	25011	41.89	0.00000
CYP2C13	cytochrome P450, family 2, subfamily c	171521	0.002	0.00000
CYP2C22	cytochrome P450, family 2, subfamily c	171518	0.37	0.00000
CYP2E1	cytochrome P450, family 2, subfamily e	25086	2.07	0.00001
CYP3A2	cytochrome P450, family 3, subfamily a	266682	0.002	0.00000
CYP3A9	cytochrome P450, family 3, subfamily a	171352	6.38	0.00000
CYP3A18	cytochrome P450, family 3, subfamily a	252931	0.06	0.00000
CYP4A12	cytochrome P450, family 4, subfamily a	266674	0.20	0.00000
CYP26	cytochrome P450, family 26	154985	11.82	0.00049
*Phase II metabolism*				
GSTA3	glutathione S-transferase, alpha 3	14859	11.80	0.00001
GSTM1	glutathione S-transferase, mu 1	24423	0.40	0.00036
GSTM2	glutathione S-transferase, mu 2	24424	0.48	0.00005
*Phase III metabolism*				
ABCB1A	ATP-binging cassette, subfamily b (MDR/TAP)	170913	2.65	0.00001
ABCC3	ATP-binging cassette, subfamily c (CFTR/MRP)	140668	10.07	0.00004
ABCC8	ATP-binging cassette, subfamily c (CFTR/MRP)	25559	0.38	0.00001
ABCC9	ATP-binging cassette, subfamily c (CFTR/MRP)	25560	0.47	0.00013

The hepatic cytochrome P450 (Cyp450) metabolizing enzymes are involved in the oxidation of the necine base of PAs with the Cyp3a's being the major enzymes catalyzing the metabolism of retronecine-based PAs to form the genotoxic pyrrolic ester DHP and *N*-oxide derivatives [1,20]. In addition, Cyp3a enzyme inducers (e.g., dexamethasone) and inhibitors (e.g., troleandomycin) cause increased and decreased riddelliine-induced DHP formation, respectively [11,21]. We observed that Cyp3a9 gene expression was increased 6-fold after riddelliine treatment which is consistent with the metabolic activation findings and suggests that Cyp3a9 is the major rat liver enzyme involved in riddelliine's metabolic activation. In human liver, it has been shown that Cyp3a4, which is equivalent to rat Cyp3a9, catalyzes the bioactivation of PAs [20]. Our finding of increase expression of Cyp2e1 is consistent with data from Gordon et al. who demonstrated that retrorsine (another of the PAs) caused increased expression of hepatic Cyp2e1 in rats [22]. The observation that the expression of many P450 genes were down-regulated may be related to the finding that many herbal/dietary constitutes form reactive intermediates capable of irreversibly inhibiting some Cyp450 enzymes [23]. The down-regulated P450 genes in this study may also contribute to decreased formation of *N*-oxide derivatives, the detoxification pathway. Thus, the biological relations of these genes related to riddelliine toxicities warrant further investigations.

The glutathione pathway plays a critical role in the detoxification of many drugs and xenobiotics. However, there is a lack of information on the types and isozymes of glutathione *S*-transferases (GST) that mediate glutathione conjugation of different PAs [1]. In this study, we observed that Gsta3 was increased 11-fold and Gstm1 and Gstm2 were decreased about 2-fold after riddelliine treatment (Table 2). These results imply that these particular types of GSTs may therefore be involved in the conjugative detoxification of riddelliine electrophiles and play an essential role in the cellular oxidative defense mechanisms. In addition, there were four ATP binding cassette transporter genes altered (2 up-regulated and 2 down-regulated) (Table 2). These phase III transporters, localized to the cell membrane, play key physiological roles in drug availability, metabolism and toxicity resulting in protection of cells and tissues against xenobiotics [24].

### Injury of liver endothelial cells

Riddelliine treatments induce relatively higher DNA adduct formation, mutation induction, and tumor incidence in the liver endothelial cell than in liver parenchymal cells [6,9,15]. In this study, the gene expression changes of a number of genes involved in the injury of liver endothelial cell were detected (Table 3). They were mainly related to cell death (Adm, F3, Tnf, Tnfrsf6, and Tnfsf10), cell movement (Edg5, Enpp2, and Il8ra), and cell-to-cell signaling and interaction (Il11, Itga4, Itga6, Lepr, and Slc7a5). We confirmed the up-regulation (2-4-fold) of previously reported Hgf, Itga4, Tnfrsf6, and Tnfsf10 [17], and also identified the novel up-regulated genes of Lepr, Prkca, Slc7a5, and Src (Table 3).

**Table 3:** Genes involved in endothelial cells altered by riddelliine treatment in liver

**Gene**	**Description**	**Locus link ID**	**Fold change**	***P*-value**
* ADM	adrenomedullin	25026	0.11	0.0010
EDG5	endothelial differentiation, G-protein-coupled receptor, 5	29415	0.19	0.0000
ENPP2	ectonucleotide pyrophosphatase/phosphodiesterase 2	84050	0.25	0.0000
F11R	F11 receptor	50848	0.43	0.0000
* F3	coagulation factor III (thromboplastin, tissue factor)	25584	0.36	0.0040
HGF	hepatocyte growth factor (hepapoietin A; scatter factor)	24446	2.46	0.0080
* IL11	interleukin 11	171040	2.38	0.0020
* IL8RA	interleukin 8 receptor, alpha	54258	2.40	0.0020
* ITGA4	integrin, alpha 4	311144	2.11	0.0020
* ITGA6	integrin, alpha 6	114517	0.35	0.0000
LEPR	leptin receptor gene-related protein; leptin receptor	16847	3.09	0.0032
* PRKCA	protein kinase C, alpha	24680	2.41	0.0000
SLC7A5	solute carrier family 7 member 5	50719	4.43	0.0040
* SRC	v-src sarcoma viral oncogene homolog (avian)	83805	3.33	0.0050
* TNF	tumor necrosis factor (TNF superfamily, member 2)	24835	0.05	0.0000
* TNFRSF6	Fas (TNF receptor superfamily, member 6)	246097	3.34	0.0010
* TNFSF10	tumor necrosis factor (ligand) superfamily, member 10	246775	2.40	0.0020

* Genes also involved in cancer development.

Leptin (Lep) has been consistently associated with angiogenesis and tumor growth. Leptin exerts its physiological action through its specific receptor (Lepr). Protein kinase C alpha (Prkca) is a serine/threonine protein kinase that has been implicated in the regulation of a variety of cellular functions in response to a diverse range of stimuli, and has recently become a target for anti-cancer therapies [25]. It has been reported that solute carrier family 7 number 5 (Slc7a5) is translated into the heavy chain of the cell surface antigen 4F2 and L-type amino acid transporter 1 and is induced by many oxidation products [26]. Src encodes non-receptor tyrosine kinases that are the intermediates of information transfer, and control pathways as diverse as cell growth, migration, death, and genome maintenance [27]. The overexpression of these genes may be responsible to the detrimental effects of riddelliine.

Also, the decreased expression of some genes, such as Adm, Edg5, and F11r, suggests a role in riddelliine-induced toxicity. The G-protein-coupled receptors were originally termed endothelial-cell-differentiation genes (EDGs) that are upregulated during endothelial cell differentiation [28]. Adrenomedullin (Adm) is a vasodilator peptide having a wide range of biological actions such as reduction of oxidative stress and inhibition of endothelial cell apoptosis [29]. The F11 receptor (F11r) plays a critical role in the function of endothelial cells and in platelet adhesion to inflamed endothelium [30]. Thus, reduction in the expression of these genes may result in less protection against oxidative stress leading to injury of liver endothelial cells.

### Genes involved in liver abnormalities

In our previous study, we determined MFs in the liver *cII *gene of Big Blue transgenic rats treated with 0.1 to 1 mg/kg riddelliine for 12 weeks, and observed increases of MF in a linear dose-dependent manner [14]. The increase in MF was consistent with dose-dependent DHP-derived DNA adduct formation [12]. Chronic exposure to 1 mg/kg of riddelliine resulted in the alteration of a number of genes involved in liver injury and abnormalities (Table 4). Significantly changed genes were divided into subsets based on functionality, and categories included cell death (e.g., Ahr, Igfbp1, Il15, and Prkcz), cellular growth and proliferation (e.g., Il7, Prkca, and Tgfa), oxidative stress (Mt1a, Nqo1, and Ren), and liver morphology (Igfbp2, Mthfd2, Pparg, and Tgfa).

**Table 4:** Genes involved in liver injury and abnormalities altered by riddelliine treatment

**Gene**	**Description**	**Locus link ID**	**Fold change**	***P*-value**
* AHR	aryl hydrocarbon receptor	25690	2.03	0.0030
* EDG5	endothelial differentiation, G-protein-coupled receptor, 5	29415	0.19	0.0000
* EGR1	early growth response 1	24330	2.13	0.0090
F7	coagulation factor VII	260320	2.22	0.0000
HGF	hepatocyte growth factor (hepapoietin A; scatter factor)	24446	2.46	0.0080
* IGFBP1	insulin-like growth factor binding protein 1	25685	3.42	0.0020
* IGFBP2	insulin-like growth factor binding protein 2	25662	15.53	0.00001
* IL11	interleukin 11	171040	2.38	0.0020
* IL15	interleukin 15	25670	2.00	0.00000
* IL7	interleukin 7	25647	0.25	0.0000
* MMP7	matrix metalloproteinase 7 (matrilysin, uterine)	25335	0.05	0.00006
* MT1A	metallothionein 1A (functional)	24567	11.10	0.0030
MTHFD2	methylenetetrahydrofolate dehydrogenase 2,	17768	0.33	0.0060
* NQO1	NAD(P)H dehydrogenase, quinone 1	24314	2.48	0.0030
* PPARG	peroxisome proliferative activated receptor, gamma	25664	0.17	0.00052
* PRKCA	protein kinase C, alpha	24680	2.41	0.0000
* PRKCZ	protein kinase C, zeta	25522	3.22	0.0020
REN	renin	24715	4.48	0.0010
* TGFA	transforming growth factor, alpha	24827	0.36	0.0000
* TNF	tumor necrosis factor (TNF superfamily, member 2)	24835	0.05	0.0000
* TNFRSF6	Fas (TNF receptor superfamily, member 6)	246097	3.34	0.0010
* TNFSF10	tumor necrosis factor (ligand) superfamily, member 10	246775	2.40	0.0020

* Genes also involved in cancer development.

The decreased expressions of Mthfd2, Pparg, Tgfa, and Tnf indicated that hepatic system development and function were harmed by riddelliine exposure, whereas the elevated Ahr, Igfbp1, Il15, Prkcz, Tnfrsf6, and Tnfsf10 were responsible for the cell death. Riddelliine treatment also resulted in 2- to 11-fold up-regulation of Mt1a, Nqo1, and Ren, suggesting the induction of oxidative stress. The metallothioneins (e.g., Mt1a), a family of proteins with antioxidant activity, are upregulated in response to zinc and oxidative stress [31]. Nqo1 gene expression is coordinately induced with other detoxifying enzyme genes in response to xenobiotics, antioxidants, oxidants, heavy metals, and radiations [32]. Renin (Ren) is involved in the renin-angiotensin-aldosterone system (RAS) which plays a major role in progressive liver fibrosis, and the blockade of the RAS could be effective in preventing fibrosis progression in chronic liver diseases [33].

### Regulation of cancer development

**Figure 5:**
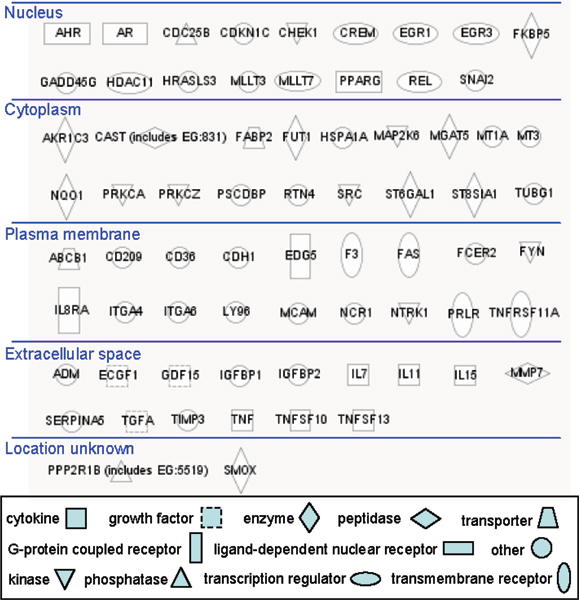
**The cellular compartments of liver proteins involved in cancer development encoded by genes whose expression was altered by riddelliine treatment**. The different categories of proteins are indicated by the shape of the symbols.

The 2-year NTP carcinogenicity study showed that riddelliine induced liver tumors in rats and male mice, lung tumors in female mice, and leukemia in rats, and that liver tumors were the cause of death for the most of these animals [5,6]. In the present study, pathway and function analysis indicated 70 genes involved in liver cancer development (Figure 5), including 17 genes coding for proteins located in nucleus, 18 genes coding for proteins in the cytoplasm, 18 genes encoding plasma membrane proteins, and 15 genes encoding proteins in the extracellular space. These significantly up- and down-regulated genes were also categorized into subgroups, including ligand-dependent nuclear receptor (Ahr, Ar, and Pparg), transcription regulator (Crem, Egr1, Egr3, Hdac11, Mllt7, and Rel), phosphatase (Cdc25b and Ppp2r1e), enzyme (Akr1c3, Fut1, Mgat5, Nqo1, St6gal1, St8sia1, Fkbp5, and Smox), kinase (Map2k6, Prkca, Prkcz, Src, Chek1, Fyn, and Ntrk1), transmembrane receptor (F3, Fas, Prlr, and Tnfrsf11a), G-protein coupled receptor (Edg5 and Il8ra), growth factor (Ecgf1, Gdf15, and Tgfa), and cytokine (Il7, Il11, Il15, Tnf, Tnfsf10, and Tnfsf13). Consequently, these genes may affect multiple cellular events that contribute to riddelliine-induced toxicological pathways.

The early gene expression changes in the carcinogenic process may involve different genes or groups of genes, depending on the carcinogen [34]. Riddelliine-induced gene expression changes appeared to involve morphology, cell death or apoptosis, growth, proliferation, and binding-related genes. Among 70 genes, 11 genes were also related to injury of liver endothelial cells (Table 3) and 18 genes were related to liver injury and abnormalities (Table 4). Riddelliine treatment increased the level of Prlr, Ncr1, Igfbp2, Mt1A, Mt3, Ppp2r1b, and Timp3 about 250, 56, 15, 11, 8, 5, and 2-fold, respectively. It has been reported that prolactin is involved in the pathogenesis of liver cirrhosis and an accumulation of the prolactin receptor (Prlr) is observed in hepatocytes damaged by cirrhosis and fibrosis [35]. The surface density of the triggering receptors (e.g., Ncr1 also called NKp46) responsible for natural killer (NK) cell-mediated cytotoxicity determines the ability of NK cells to kill susceptible target cells [36]. The dramatic increase in Ncr1 may, therefore, indicate an increase in cells susceptible to cell-mediated toxicity. Insulin-like growth factor-binding proteins (IGFBPs) are important modulators of IGF actions, and overexpression of Igfbp2 is observed in a variety of pathological conditions. In addition, Igfbp2 is expressed in many malignant tissues including liver, and Igfbp2 appears to be a suitable marker for the evaluation of the serological status of HCC patients [37]. Metallothioneins (MT) are a group of low-molecular weight and cysteine rich intracellular proteins. The expression and induction of these genes (e.g., Mt1a and Mt3) have been associated with protection against DNA damage, oxidative stress and apoptosis. A number of studies have shown an increased expression of MT in various human tumors including hepatoma [38]. Ppp2r1b has been implicated as a tumor suppressor gene, and somatic alterations of Ppp2r1b have been detected in several cancers. Most recently, Chou et al. reported that aberrant transcripts of Ppp2r1b might be associated with the development of HCC [39]. Tumor-necrosis factor (TNF) is a pleiotropic cytokine that triggers physiological and pathological responses in several organs. TIMP3 is a crucial innate negative regulator of TNF in both tissue homeostasis and tissue response to injury [40]. Thus, the overexpression of these genes supports a critical role of them in the toxicology of riddelliine.

## Conclusion

The present study represents the first comprehensive in vivo examination of the chronic transcriptional response of the liver to riddelliine exposure. The available evidence on the metabolism and target-tissue specificity for riddelliine's tumorigenesis suggests that active metabolites of riddelliine interact with endothelial cells in the liver, which causes cell toxicity, followed by compensatory proliferation of DNA-damaged endothelial cells, 'fixation' of the adducts into mutations in these cells, and eventual development of hemangiosarcoma and HCC. We have identified 919 genes in the livers of riddelliine-treated rats that were differentially expressed and related to these physiological and pathological outcomes. Relating the gene expression changes to phenotypic anchors such as metabolism, injury of liver endothelial cells, liver abnormalities, and cancer development, has helped in the interpretation of these data. Although the significance of all of the hundreds of gene expression changes is not fully understood, the genome-wide global information obtained herein will contribute to an improved understanding of the molecular alterations that occur after exposure to riddelliine, and provide further insight into the mechanisms involved in toxicity and carcinogenesis.

## Materials and methods

### Chemical and animals

Riddelliine (>97% pure by reversed-phase HPLC analysis) was obtained from the NTP and dissolved in 0.9% sodium chloride. Female Big Blue Fisher 344 transgenic rats were obtained from Taconic Laboratories (Germantown, NY) through purchase from Stratagene (La Jolla, CA). All animal procedures followed the recommendations of the NCTR Institutional Animal Care and Use Committee for the handling, maintenance, treatment, and sacrifice.

### Riddelliine treatment

The treatment schedule was based on the preliminary results from the NTP two-year chronic tumorigenicity bioassay [5]. Six-week-old Big Blue rats were treated with riddelliine at the dose of 1 mg/kg body weight by gavage five times a week for 12 weeks. Vehicle control rats were gavaged with 0.9% sodium chloride. Six rats from treatment and control groups were sacrificed one day after the last treatment. The livers were isolated, frozen quickly in liquid nitrogen, and stored at -80°C. Tumors had not developed at the 12 week sacrifice time so that gene expression changes reflected early events in the carcinogenesis process.

### RNA isolation and quality control

Total RNA was isolated from liver tissues of 6 control and 6 riddelliine-treated rats using an RNeasy system (Qiagen, Chatsworth, CA). The yield of the extracted RNA was determined spectrophotometrically by measuring the optical density at 260 nm. The purity and quality of extracted RNA were evaluated using the RNA 6000 LabChip and Agilent 2100 Bioanalyzer (Agilent Technologies, Palo Alto, CA). RNA samples with RNA integrity numbers (RINs) greater than 8.5 were used for microarray experiments performed using Applied Biosystems' Rat Genome Survey Microarray platform, which is a one channel microarray with chemiluminescence detection, and contains 26,857 probes (60-mer) for the interrogation of 27,088 genes and 1592 controls that track system performance through each experiment.

### Preparation of digoxigenin labeled in vitro transcribed cRNA

All RNA targets were labeled using the Applied Biosystems RT-IVT Labeling Kit Version 2.0. Briefly, 1.5 μg of total RNA was reverse transcribed via 2 h incubation at 42°C with ArrayScript RT enzyme (Ambion, Austin, TX) and oligo dT-T7 primer. Double stranded cDNA was produced following 2 h incubation with *E. coli *DNA polymerase and RNase H at 16°C. Double-stranded cDNA was purified according to the RT-IVT kit protocol. In vitro transcription was performed by incubation of the cDNA product with T7 RNA polymerase, 0.75 mM Digoxigenin-11-UTP (Roche Applied Science, Indianapolis, IN) and all other NTPs for 9 h. Labeled cRNA was purified according to the RT-IVT kit protocol and analyzed for quality and quantity using standard UV spectrometry and the Bioanalyzer.

### Hybridization of labeled cRNA to microarrays and microarray imaging

Digoxigenin labeled cRNA targets were hybridized to Applied Biosystems Rat Whole Genome Survey Microarrays using the Applied Biosystems Chemiluminescent Detection Kit. Briefly, 15 μg of labeled cRNA targets were fragmented via incubation with fragmentation buffer provided in the kit for 30 min at 60°C. Fragmented targets were hybridized to microarrays during a 16 h incubation at 55°C with buffers and reagents from the Chemiluminescent Detection Kit. Post-hybridization washes and anti-Digoxigenin-Alkaline Phosphatase binding were performed according to the protocol of the kit. Chemiluminescence detection, image acquisition and analysis were performed using Applied Biosystems Chemiluminescence Detection Kit and Applied Biosystems 1700 Chemiluminescent Microarray Analyzer following the manufacturer's protocols. Images were auto-gridded and the chemiluminescent signals were quantified, corrected for background, and finally, spot- and spatially-normalized using the Applied Biosystems 1700 Chemiluminescent Microarray Analyzer software version 1.1.

### Microarray data analysis

Raw microarray intensity data from the Applied Biosystems' Rat Genome Survey Microarray were normalized with Quantile normalization which is recommended by the manufacturer. The normalized data were then input to ArrayTrack, a software system developed by the FDA's National Center for Toxicological Research for the management, analysis, visualization and interpretation of microarray data [41]. Chemiluminescent signals from 1529 control probes that track system performance through each experiment were not used in normalization. The identification of differentially expressed genes based on fold-change and *t*-tests cutoffs, and Principal Component Analysis were conducted within ArrayTrack. Ingenuity Pathway Analysis (Mountain View, CA) was used for network and function analysis.

## Competing interests

The authors declare that they have no competing interests.

## Authors' contributions

NM performed the animal treatment and was involved in the analysis of microarray data, and wrote the manuscript. LG, QL, and JCF helped conceive the experiments, analyze the data, and write the manuscript. TC was involved in designing the experiment and writing the manuscript. All authors approved the final version of manuscript.

## References

[B1] Fu PP, Xia Q, Lin G, Chou MW (2004). Pyrrolizidine alkaloids – genotoxicity, metabolism enzymes, metabolic activation, and mechanisms. Drug Metab Rev.

[B2] International Programme on Chemical Safety (1989). Pyrrolizidine Alkaloids Health and safety Guide. Health and Safety Criteria Guide.

[B3] Xia Q, Chou MW, Edgar JA, Doerge DR, Fu PP (2006). Formation of DHP-derived DNA adducts from metabolic activation of the prototype heliotridine-type pyrrolizidine alkaloid, lasiocarpine. Cancer Lett.

[B4] Fu PP, Chou MW, Xia Q, Yang YC, Yan J, Doerge DR, Chan PC (2001). Genotoxic pyrrolizidine alkaloids and pyrrolizidine alkaloid N-oxides - mechanisms leading to DNA adduct formation and tumorigenicity. Environ Carcinog Ecotoxicol Rev.

[B5] National Toxicology Program (2003). Toxicology and carcinogenesis studies of riddelliine (CAS No. 23246-96-0) in F344/N rats and B6C3F1 mice (gavage studies). Natl Toxicol Program Tech Rep Ser.

[B6] Chan PC, Haseman JK, Prejean JD, Nyska A (2003). Toxicity and carcinogenicity of riddelliine in rats and mice. Toxicol Lett.

[B7] Chan PC, Mahler J, Bucher JR, Travlos GS, Reid JB (1994). Toxicity and carcinogenicity of riddelliine following 13 weeks of treatment to rats and mice. Toxicon.

[B8] Chou MW, Wang YP, Yan J, Yang YC, Beger RD, Williams LD, Doerge DR, Fu PP (2003). Riddelliine N-oxide is a phytochemical and mammalian metabolite with genotoxic activity that is comparable to the parent pyrrolizidine alkaloid riddelliine. Toxicol Lett.

[B9] Chou MW, Yan J, Nichols J, Xia Q, Beland FA, Chan PC, Fu PP (2003). Correlation of DNA adduct formation and riddelliine-induced liver tumorigenesis in F344 rats and B6C3F(1) mice. Cancer Lett.

[B10] Williams L, Chou MW, Yan J, Young JF, Chan PC, Doerge DR (2002). Toxicokinetics of riddelliine, a carcinogenic pyrrolizidine alkaloid, and metabolites in rats and mice. Toxicol Appl Pharmacol.

[B11] Xia Q, Chou MW, Kadlubar FF, Chan PC, Fu PP (2003). Human liver microsomal metabolism and DNA adduct formation of the tumorigenic pyrrolizidine alkaloid, riddelliine. Chem Res Toxicol.

[B12] Yang YC, Yan J, Doerge DR, Chan PC, Fu PP, Chou MW (2001). Metabolic activation of the tumorigenic pyrrolizidine alkaloid, riddelliine, leading to DNA adduct formation in vivo. Chem Res Toxicol.

[B13] Chou MW, Jian Y, Williams LD, Xia Q, Churchwell M, Doerge DR, Fu PP (2003). Identification of DNA adducts derived from riddelliine, a carcinogenic pyrrolizidine alkaloid. Chem Res Toxicol.

[B14] Mei N, Heflich RH, Chou MW, Chen T (2004). Mutations induced by the carcinogenic pyrrolizidine alkaloid riddelliine in the liver *cII *gene of transgenic Big Blue rats. Chem Res Toxicol.

[B15] Mei N, Chou MW, Fu PP, Heflich RH, Chen T (2004). Differential mutagenicity of riddelliine in liver endothelial and parenchymal cells of transgenic Big Blue rats. Cancer Lett.

[B16] Guo L, Lobenhofer EK, Wang C, Shippy R, Harris SC, Zhang L, Mei N, Chen T, Herman D, Goodsaid FM (2006). Rat toxicogenomic study reveals analytical consistency across microarray platforms. Nat Biotechnol.

[B17] Mei N, Guo L, Zhang L, Shi L, Sun YA, Fung C, Moland CL, Dial SL, Fuscoe JC, Chen T (2006). Analysis of gene expression changes in relation to toxicity and tumorigenesis in the livers of Big Blue transgenic rats fed comfrey (*Symphytum officinale*). BMC Bioinformatics.

[B18] Chen T, Guo L, Zhang L, Shi L, Fang H, Sun Y, Fuscoe JC, Mei N (2006). Gene expression profiles distinguish the carcinogenic effects of aristolochic acid in target (kidney) and non-target (liver) tissues in rats. BMC Bioinformatics.

[B19] Yang Y, Yan J, Churchwell M, Beger R, Chan P, Doerge DR, Fu PP, Chou MW (2001). Development of a (32)P-postlabeling/HPLC method for detection of dehydroretronecine-derived DNA adducts in vivo and in vitro. Chem Res Toxicol.

[B20] Miranda CL, Reed RL, Guengerich FP, Buhler DR (1991). Role of cytochrome P450IIIA4 in the metabolism of the pyrrolizidine alkaloid senecionine in human liver. Carcinogenesis.

[B21] Wang YP, Yan J, Beger RD, Fu PP, Chou MW (2005). Metabolic activation of the tumorigenic pyrrolizidine alkaloid, monocrotaline, leading to DNA adduct formation in vivo. Cancer Lett.

[B22] Gordon GJ, Coleman WB, Grisham JW (2000). Induction of cytochrome P450 enzymes in the livers of rats treated with the pyrrolizidine alkaloid retrorsine. Exp Mol Pathol.

[B23] Zhou S, Koh HL, Gao Y, Gong ZY, Lee EJ (2004). Herbal bioactivation: the good, the bad and the ugly. Life Sci.

[B24] Klaassen CD (2002). Xenobiotic transporters: another protective mechanism for chemicals. Int J Toxicol.

[B25] Michie AM, Nakagawa R (2005). The link between PKCalpha regulation and cellular transformation. Immunol Lett.

[B26] Takabe W, Kanai Y, Chairoungdua A, Shibata N, Toi S, Kobayashi M, Kodama T, Noguchi N (2004). Lysophosphatidylcholine enhances cytokine production of endothelial cells via induction of L-type amino acid transporter 1 and cell surface antigen 4F2. Arterioscler Thromb Vasc Biol.

[B27] Russello SV, Shore SK (2004). SRC in human carcinogenesis. Front Biosci.

[B28] Luquain C, Sciorra VA, Morris AJ (2003). Lysophosphatidic acid signaling: how a small lipid does big things. Trends Biochem Sci.

[B29] Kato J, Tsuruda T, Kita T, Kitamura K, Eto T (2005). Adrenomedullin: a protective factor for blood vessels. Arterioscler Thromb Vasc Biol.

[B30] Sobocki T, Sobocka MB, Babinska A, Ehrlich YH, Banerjee P, Kornecki E (2006). Genomic structure, organization and promoter analysis of the human F11R/F11 receptor/junctional adhesion molecule-1/JAM-A. Gene.

[B31] Chung MJ, Hogstrand C, Lee SJ (2006). Cytotoxicity of nitric oxide is alleviated by zinc-mediated expression of antioxidant genes. Exp Biol Med (Maywood).

[B32] Jaiswal AK (2000). Regulation of genes encoding NAD(P)H:quinone oxidoreductases. Free Radic Biol Med.

[B33] Bataller R, Sancho-Bru P, Gines P, Brenner DA (2005). Liver fibrogenesis: a new role for the renin-angiotensin system. Antioxid Redox Signal.

[B34] Iida M, Anna CH, Holliday WM, Collins JB, Cunningham ML, Sills RC, Devereux TR (2005). Unique patterns of gene expression changes in liver after treatment of mice for 2 weeks with different known carcinogens and non-carcinogens. Carcinogenesis.

[B35] Simon-Holtorf J, Monig H, Klomp HJ, Reinecke-Luthge A, Folsch UR, Kloehn S (2006). Expression and distribution of prolactin receptor in normal, fibrotic, and cirrhotic human liver. Exp Clin Endocrinol Diabetes.

[B36] Djeu JY, Jiang K, Wei S (2002). A view to a kill: signals triggering cytotoxicity. Clin Cancer Res.

[B37] Ranke MB, Maier KP, Schweizer R, Stadler B, Schleicher S, Elmlinger MW, Flehmig B (2003). Pilot study of elevated levels of insulin-like growth factor-binding protein-2 as indicators of hepatocellular carcinoma. Horm Res.

[B38] Cherian MG, Jayasurya A, Bay BH (2003). Metallothioneins in human tumors and potential roles in carcinogenesis. Mutat Res.

[B39] Chou HC, Chen CH, Lee HS, Lee CZ, Huang GT, Yang PM, Lee PH, Sheu JC (2007). Alterations of tumour suppressor gene PPP2R1B in hepatocellular carcinoma. Cancer Lett.

[B40] Mohammed FF, Smookler DS, Taylor SE, Fingleton B, Kassiri Z, Sanchez OH, English JL, Matrisian LM, Au B, Yeh WC (2004). Abnormal TNF activity in Timp3-/- mice leads to chronic hepatic inflammation and failure of liver regeneration. Nat Genet.

[B41] Tong W, Cao X, Harris S, Sun H, Fang H, Fuscoe J, Harris A, Hong H, Xie Q, Perkins R (2003). ArrayTrack – supporting toxicogenomic research at the U.S. Food and Drug Administration National Center for Toxicological Research. Environ Health Perspect.

